# Dielectrophoretic analysis of the impact of isopropyl alcohol on the electric polarisability of *Escherichia coli* whole-cells

**DOI:** 10.1007/s00216-020-02451-9

**Published:** 2020-03-11

**Authors:** Miriam S. Epping, Severin Wedde, Armin Grundmann, Marco Radukic, Harald Gröger, Anke Hummel, Martina Viefhues

**Affiliations:** 1 Experimental Biophysics and Applied Nanosciences, Department of Physics, Bielefeld University, 33615 Bielefeld, Germany; 2 Industrial Organic Chemistry and Biotechnology, Department of Chemistry, Bielefeld University, 33615 Bielefeld, Germany; 3 Present Address: Fermentation Engineering, Department of Technology, Bielefeld University, 33615 Bielefeld, Germany; 4 Present Address: Cellular and Molecular Biotechnology, Department of Technology, Bielefeld University, 33615 Bielefeld, Germany

**Keywords:** Microfluidics, Whole-cell biocatalyst, Dielectrophoresis analysis, UV-Vis absorbance analysis, Cofactor leaching

## Abstract

Whole-cell biocatalysts are versatile tools in (industrial) production processes; though, the effects that impact the efficiency are not fully understood yet. One main factor that affects whole-cell biocatalysts is the surrounding medium, which often consists of organic solvents due to low solubility of substrates in aqueous solutions. It is expected that organic solvents change the biophysical and biochemical properties of the whole-cell biocatalysts, e.g. by permeabilising the cell membrane, and thus analysis of these effects is of high importance. In this work, we present an analysis method to study the impact of organic solvents on whole-cell biocatalysts by means of dielectrophoresis. For instance, we evaluate the changes of the characteristic dielectrophoretic trapping ratio induced by incubation of *Escherichia coli*, serving as a model system, in an aqueous medium containing isopropyl alcohol. Therefore, we could evaluate the impact on the electric polarisability of the cells. For this purpose, a special microchannel device was designed and *Escherichia coli* cells were genetically modified to reliably synthesise a green fluorescent protein. We could demonstrate that our method was capable of revealing different responses to small changes in isopropyl alcohol concentration and incubation duration. Complementary spectrophotometric UV-Vis (ultraviolet-visible light) absorbance analysis of released NAD(P)^+^/NAD(P)H cofactor and proteins confirmed our results. Based on our results, we discuss the biophysical effects taking place during incubation.

**Electronic supplementary material:**

The online version of this article (10.1007/s00216-020-02451-9) contains supplementary material, which is available to authorised users.

**Figure:**
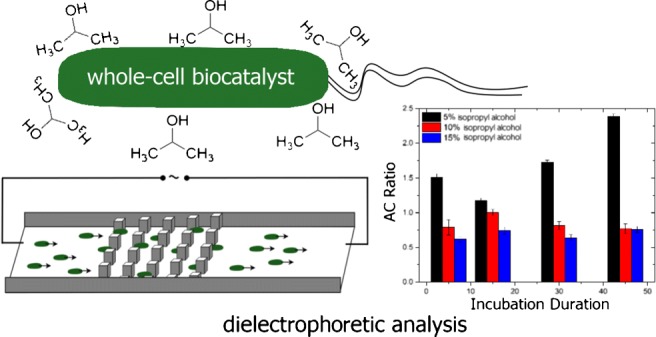
Graphical abstract

## Introduction

*Escherichia coli* (*E. coli*) cells are versatile organisms that are often used as host organisms in the construction of whole-cell biocatalysts. Their stable and well-controlled microenvironment yields very good conditions for enzymes; the intracellular matrix further stabilises enzymes. Moreover, *E. coli* consist of numerous intrinsic cofactors that are important for a large number of industrially used enzymes, e.g. alcohol dehydrogenase or monooxygenase. Furthermore, *E. coli* is considered a useful host as it enables a high overexpression of recombinant proteins and is easy to handle and to manipulate, and the whole-cells are the most readily available form of the recombinant enzyme where no further preparation step is necessary. Therefore, biocatalysis in whole-cell biocatalysts can be more stable and reliable as in batch reactors with free enzymes. Thus, about 60% of the industrial biocatalytic reactions are performed with whole-cell biocatalysts as reported in 2002 by Straathof et al. [1].

The well-balanced (electro)physiology in *E. coli* might play an important role for efficient biocatalysis. Thus, if the membrane is permeabilised, electrolytes, cofactors, and other cell compounds leak out of the cell and the biocatalytic efficiency might decrease [2]. Cofactor release can be due to the complex reaction media used in biocatalytic processes. For example, several substrates are insoluble in aqueous solutions. Thus, frequently, the solubility is increased by the addition of organic solvents, either as aqueous-organic one- or two-phase system. It is assumed that the organic solvents change the permeability of the cell membrane so that cofactors can be released out of the cell. For some substrates, the opposite applies; i.e., the native cell membrane is impermeable for them. In that case, the membrane needs to be permeabilised for the respective substrate. That can be done by genetic engineering, e.g. by introducing substrate-specific channels in the membrane or freeze-thaw cycles.

The impact of organic solvents on the cell membrane is not completely understood and needs further investigations [3]. Additionally, in some cases, whole-cell biocatalysts are (specifically) permeabilised such that the substrate can diffuse into the cell. In both cases, the effects on the cell membrane must be understood to improve biotransformation processes. Several studies have been performed to determine the impact of chemicals and the logP value, i.e. the logarithm of the octanol-water partition, is a value that was used for the characterisation [3]. But this provides only a general tendency of the impact on whole-cell biocatalytic reactions. Other methods were based on growth and survival assays, which take several hours up to days, and thus are very time-consuming [4, 5]. Anrade et al. and Reimer et al. investigated the impact of immobilisation matrices and cofactor release of *E. coli* on (bio)catalytic reactions. They determined the impact of the cofactor loss indirectly by monitoring the enzymatic activity [6, 7].

Changes in the dielectricity or conductivity, as for dead cells, membrane truncation or different cell phases, affect the electrical impedance and the dielectrophoretic migration, and thus can be detected [8–11]. Dielectrophresis is the migration of an electrically polarisable object in an inhomogeneous electric field. The dielectrophoretic force is proportional to the polarisability of the respective object [12]. Zhivkov and Gyurova investigated the polarisability of cells and the impact of ethanol. They discussed the mechanisms of polarisation with the Maxwell-Wagner theory versus charge dependent polarisation mechanism [13–16]. Zhu et al. exploited impedance measurements to gain information about the electric cell polarisability or the cell membrane, i.e. if the ion channels were open or closed. They monitored the changes during the cell cycle and could distinguish the different cell status [9]. Several groups exploited the dielectrophoretic response of various cells for characterisation and separation purposes [17–23]. Jones et al. reported that *E. coli* of different serotypes could be discriminated by their dielectric response [19]. Thus, already small changes in the cellular (bio)chemical composition have significant impact on the electric properties. Cells were analysed with dielectrophoresis (DEP) with respect to their Gram stain [24], if the cells are dead or alive was dielectrophoretically investigated [21] and different cell types were distinguished by DEP [25]. Thus, the dielectric properties of the cells are suitable criteria to characterise cells.

In this work, we developed a new dielectrophoresis-based analysis of the impact of organic solvents on whole-cell biocatalysts, which could contribute to a more efficient process development for such biotransformations. Isopropyl alcohol was chosen as an example for organic solvents as it can be applied in whole-cell reactions with dehydrogenases for regeneration of NAD(P)^+^/NAD(P)H cofactor. The migration in a dielectrophoretic potential landscape was exploited to evaluate changes of intrinsic properties.

## Materials and methods

### Construction of the plasmid for expression of green fluorescent protein

All chemicals were obtained from Carl Roth or VWR, and all molecular biological tools were from Thermo Fisher Scientific, unless otherwise stated. The gene of the fluorescence protein SuperfolderGFP [26] was codon-optimised with the GeneOptimizer^TM^ algorithm [27] for expression in *Escherichia coli* and obtained as synthetic gene (Thermo Fisher Scientific).

The plasmid pET21T5_ompA-co-SuperfolderGFP(-), that was used for expression of the green fluorescent protein (GFP), was constructed based on the plasmid pET21a(+) (Novagen/Merck) by exchange of the T7 promoter by the constitutive T5 promoter that can be recognised by *E. coli* enabling recombinant protein expression without induction resulting in the plasmid pET21aT5 (for details of the cloning strategy and the molecular biological steps as well as detailed vector map and sequence information, see Electronic Supplementary Material).

### Preparation of *Escherichia coli* expressing GFP

Chemically competent *Escherichia coli* DH5*α* cells (Thermo Fisher Scientific) were transformed with the plasmid pET21T5_co-SuperfolderfGFP(-) by heat shock standard method [28]. LB medium (30 mL, 10 g/L NaCl, 10 g/L peptone, 5 g/L yeast extract (all from Carl Roth GmbH + Co. KG), pH 7.4) [29] supplemented with carbenicillin (100 mg/mL) was inoculated with an overnight culture from a freshly transformed single colony to OD_600nm_ = 0.1 and grown at 37 °C and 180 rpm (Infors Ecotron) in a 300-mL shaking flask until OD_600nm_ reached 1.2–1.4. Induction of expression of GFP was not necessary as the promoter enabled constitutive expression. Cells were harvested by centrifugation (4000 × *g*, 4 °C, 15 min), washed once with resuspension buffer (50 mM Tris, 10 mM EDTA, pH 7.5 with HCl) and stored at 4–8 °C until further use for a maximum of 3 days.

### Microfluidic analysis

**Fig. 1:**
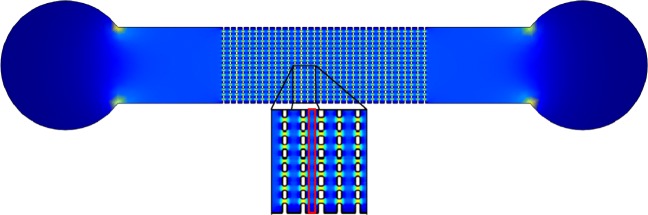
Top view sketch of device with electric field strength (colour-coded). The DEP force is generated in the array of insulating posts. The vertical gaps between two posts were 10 *μ*m in size. The horizontal distance between two gaps was 20 *μ*m. Thus, the periodicity was 30 *μ*m. Inset: zoom to posts with area in which the fluorescence intensity was determined indicated (red box)

A linear microfluidic channel with insulating posts was used in the experiments (see Fig. 1). The channel had a height, width, and length of 5 *μ*m, 380 *μ*m, and 1 cm, respectively. The gaps defined by the posts had a width and length of 10 × 10 *μ*m^2^. The device consists of 110 columns of posts with 20-*μ*m distance in between. The microfluidic chip was made by poly(dimethylsiloxane) (PDMS) soft lithography as described elsewhere [30]. We used MilliQ water with 0.3 M sucrose to prevent osmotic pressure. Pluronic (F_108_ BASF, Germany) was used in all buffers as dynamic surface coating to reduce unspecific adhesion of the cells to the chip [31].

After harvesting of the *E. coli* (see Section “Preparation of *Escherichia coli* expressing GFP”), cells (OD_600nm_ = 1.4) were incubated in isopropyl alcohol of varying concentrations, i.e. 5%, 10%, and 15% (v/v) for different incubation durations, i.e. 5 min, 15 min, 30 min, and 45 min. Afterward, the incubation buffer was exchanged with sucrose solution (0.3 M in MilliQ water). For that purpose, the cells were centrifugated at 9300 × *g* for 1 min and the supernatant was disposed. The cells were carefully resuspended in 0.3 M sucrose buffer. The centrifugation and resuspension was performed 5 times to completely remove the isopropyl alcohol before the dielectrophoretic migration behaviour was analysed.

**Fig. 2:**
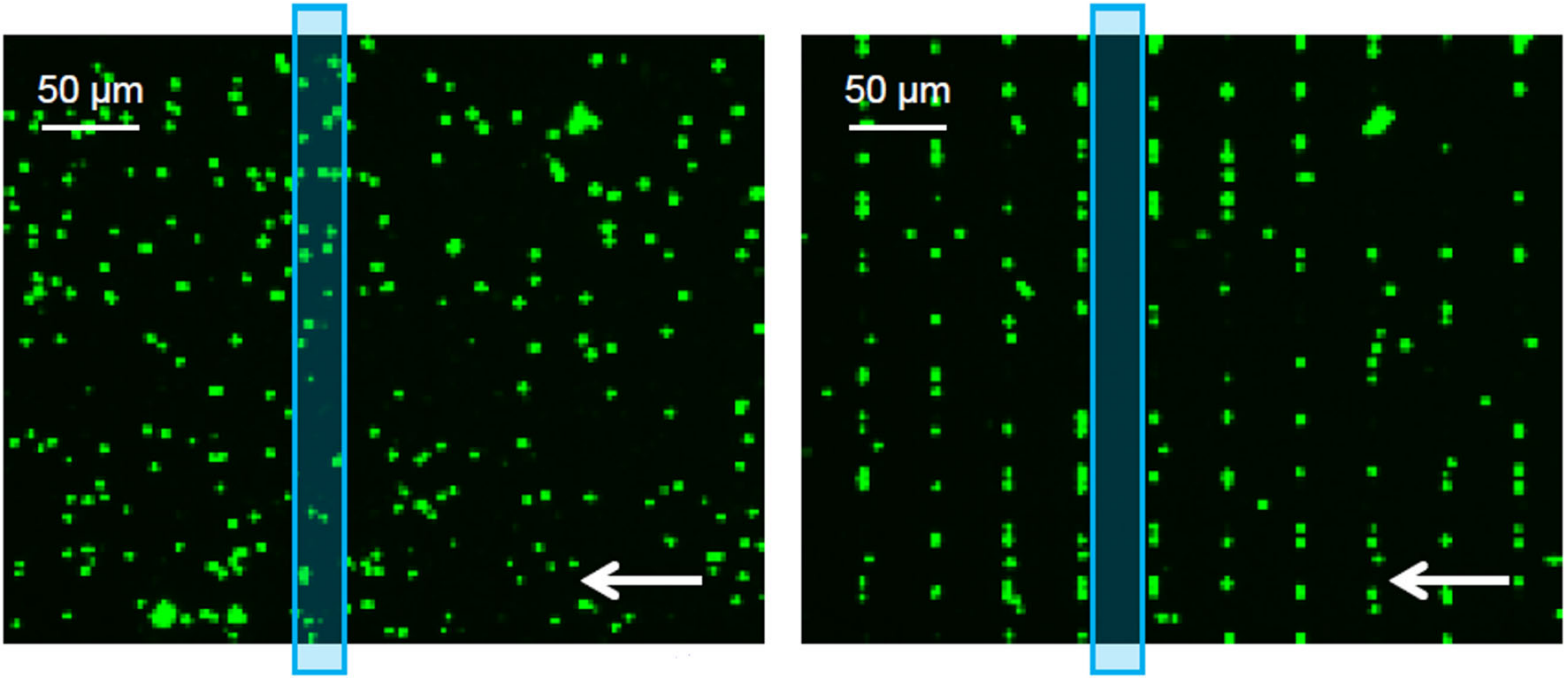
Measurement process. The fluorescence intensity is determined in the region of interest (ROI, blue rectangle). (Left) |*U*_AC_| = 0 the *E. coli* are not trapped. (Right) |*U*_AC_| = 500 V the *E. coli* are trapped between the posts, i.e. exhibiting positive DEP. The direction of flow is indicated by the white arrows

For the DEP analysis, the *E. coli* were continuously flushed through the microfluidic channel, consisting of an array of insulating posts, by applying 0.5 mbar, which results in a flow rate of about 2 nL/min. Then, sinusoidal AC voltages, frequency of 1 kHz and stepwise increased from 50 to 900 V, were applied to platinum electrodes submerged in the two reservoirs yielding dielectrophoretic forces in the gaps between the insulating posts [32]. The fluorescence intensity was evaluated in the region between the post rows (Fig. 2), i.e. in the region with homogeneous electric field. Thus, for sufficiently high AC voltages, no cells were in the region of interest (see also Section “Theory”). The experimental setup is described in our previous work [33]. Additionally, a MFCS pump (Fluigent, Germany) was used to move the fluids through the microfluidic channel.

### UV-Vis analysis

*E. coli* cells prepared as described in Section “Preparation of *Escherichia coli* expressing GFP” were utilised for UV-Vis experiments on the same day after cultivation. The cell pellet was carefully resuspended in a mixture of sucrose solution and isopropyl alcohol in varying amounts to yield a cell density of OD_600nm_ = 20. Isopropyl alcohol was added to different final amounts (0%, 5%, 10%, 15%, 20%, 25%, 40% (v/v)) and sucrose concentration was kept constant (0.3 M). The experimental setup was chosen in a way that the cells were kept on ice in sucrose solution to 0.3 M until use, distributed into aliquots (400 *μ* L final volume consisting of 200 *μ* L of concentrated cell suspension with OD_600nm_ 40 in sucrose (final concentration 0.3 M), 40 *μ* L of sucrose stock solution (1.5 M yielding overall 0.3 M final sucrose concentration) and 160 *μ* L of varying amounts of MilliQ water and isopropyl alcohol) in 1.5-mL plastic reaction tubes, and addition of isopropyl alcohol marked *t*_0_ = 0 min as start of incubation. The reaction tubes were incubated (25 °C, 1000 rpm) for different times (5 min, 15 min, 30 min, 45 min). All reactions were performed in quituplicates. After incubation, the cells were separated from the supernatant by centrifugation (5000 × *g*, 4 °C, 5 min) and a part of the supernatant (200 *μ* L) was transferred into 96-well microtiterplates. Amount of NAD(P)^+^/NAD(P)H cofactor released from the cells was determined by detection of the absorption at 340 nm (Microtiterplate Reader Spark 10M, Tecan), and quantified by a specific assay (Total NADP and NADPH assay kit and Total NAD and NADH assay kit, Abcam). The amount of protein released from the cells was determined by Bradford assay against bovine serum albumin as standard at 595 nm [34]. The blank contained 0.3 M sucrose; in preliminary experiments, the impact of isopropyl alcohol on the assay was excluded.

For the experimental determination of the maximum content of NAD(P)^+^/NAD(P)H cofactor or protein in the cells, a sample (quintuplicate determination) prepared as described above not pretreated with isopropyl alcohol was disrupted by sonication (2 × 3 min at 50% cycle, 20% power (Bandelin Sonoplus), on ice, 5 min on ice in between treatments). The cell debris was pelleted by centrifugation (10 min, 20,000 × *g*, 4 °C) and the supernatant (200 *μ*L) was studied as described above to determine NAD(P)^+^/NAD(P)H cofactor and protein content.

## Theory

Dielectrophoresis is the migration of a polarisable object in an inhomogeneous electric field [12]. The dielectrophoretic potential is given by [35]

1 $$ W_{\text{DEP}}=\alpha\cdot\mathbf{E}^{2} $$

with *α* polarisability of the object and **E** the electric field.

The polarisability depends on the surrounding medium and material constants of the sample, e.g. the dielectric permittivity and conductivity [36]. Therefore, dielectrophoresis is a sensitive method that is capable to study changes in the cell membrane and detects differences in the cell’s electrophysiology [15, 17–22].

In this work, the impact of organic solvents on *E. coli* was studied based on the migration in a dielectrophoretic potential landscape. In general, the migration of a polarisable object in an inhomogeneous electric field can be described as a superposition of linear and non-linear responses to applied forces. With $U_{\text {DC}}^{2}<<U_{\text {AC}}^{2}$, the migration can be described by [35]

2 $$ \bar{\mathbf{u}}=(\mu_{\text{ep}}+\mu_{\text{eo}})\mathbf{E}_{\text{DC}}+\mu_{\text{DEP}}\mathbf{\nabla}\bar{\mathbf{E}}^{2} $$

with $\bar {\mathbf {u}}$ mean velocity, *μ*_ep,eo,DEP_ the electrophoretic, electroosmotic and dielectrophoretic mobilities, respectively.

Here, the *E. coli* were driven by hydrostatic pressure through the dielectrophoretic potential landscape. Thus, the linear electrokinetic terms can be replaced by a Hagen-Poiseuille velocity term. Samples are trapped in a DEP potential if the DEP force overcomes the thermal motion of the sample, i.e. diffusion, linear electrokinetic migration and pressure driven flow [32]. Since the DEP potential depth is proportional to the polarisability, the AC voltage needed to trap cells is strongly correlated to the cell’s electric properties, i.e. conductivity and permittivity [36].

## Results and discussion

### Dielectrophoresis analysis

The concept of the DEP analysis in this work is based upon the assumption that the dielectrophoretic migration behaviour of *E. coli* changes if the electrophysiology of the cell is changed by the organic solvents. The changes can apply to the membrane; e.g., the fluidity can change if organic solvent molecules are incorporated into the membrane [37–39] or the membrane gets disorganised such that (small) charged molecules can flow out of the cell. Based on these assumptions, we determined the characteristic migration behaviour of *E. coli* in a dielectrophoretic potential landscape and compared the migration with that of *E. coli* that were incubated in an aqueous medium containing isopropyl alcohol.

**Fig. 3:**
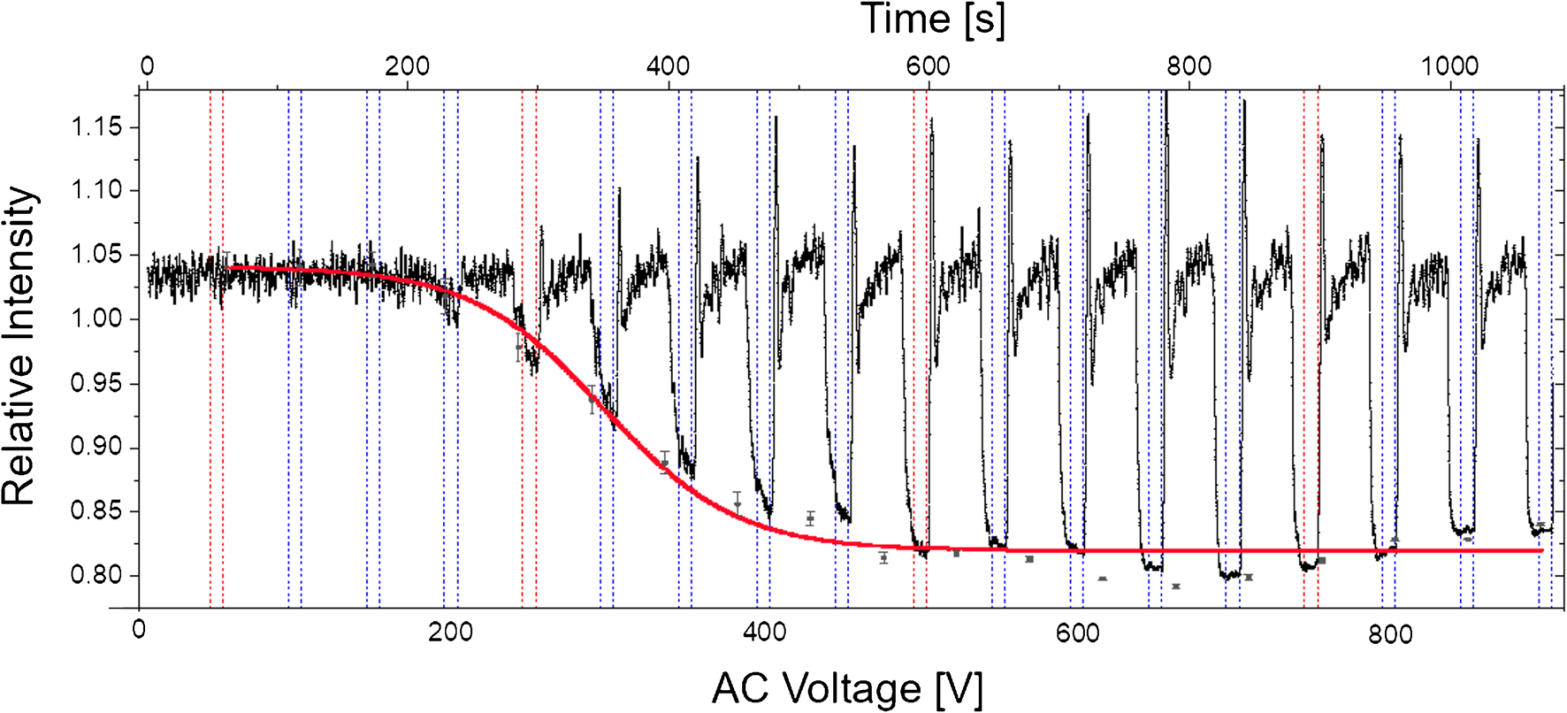
Relative fluorescence intensity during experiment. The relative intensity was determined by dividing the mean fluorescence intensity determined in the region between the post rows by the mean fluorescence of the whole picture. The black line is the relative intensity, the dots are the mean values determined during DEP trapping (indicated by the vertical dotted lines), and the red line is the fit with the Boltzman function to determine the AC voltage at which 50% of cells were trapped

First, a sufficient characterisation protocol was developed to determine the reference migration behaviour of the cells to be compared with those incubated in an aqueous medium containing isopropyl alcohol. For that purpose, we used an array of insulating posts and flushed the *E. coli* through that array at controlled velocity by pressure-driven flow. A dielectrophoretic force was superimposed by applying an AC voltage for 30 s while monitoring the migration of the *E. coli* (see Section “Microfluidic analysis”). We determined the characteristic AC voltage that was needed to dielectrophoretically trap 50% of the *E. coli*. The trapping ratio was determined by evaluation of the fluorescence intensity measured in defined regions of interest (ROI, Fig. 2). The intensity in the ROI decreased as *U*_AC_ increases, because the *E. coli* were trapped in the small gaps between the posts, due to positive dielectrophoresis. The fluorescence intensity was plotted against the applied AC voltage, the data were fitted, with the logistic Boltzman function and the voltage at *I*_50*%*_ was determined (Fig. 3).

**Fig. 4:**
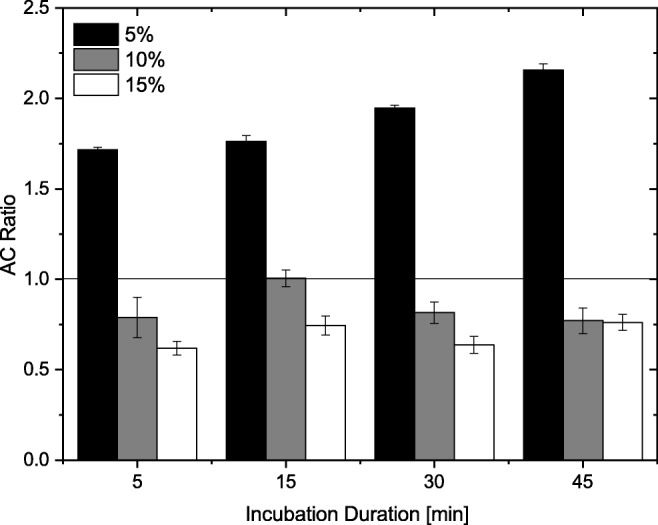
Change of characteristic AC voltage, i.e. at inflection point, dependent on incubation with isopropyl alcohol. The cells were incubated in isopropyl alcohol of varying concentrations, 5%, 10%, and 15%, and the DEP response was determined after 5, 15, 30, and 45 min

After setting up the evaluation concept, we investigated the impact of isopropyl alcohol on *E. coli*. We varied the concentration of isopropyl alcohol, 5%, 10%, and 15% (v/v), and the incubation duration, 5 min, 15 min, 30 min, and 45 min, to study which effects occur. During the experiments, we observed variations in the absolute AC voltages that were due to inter chip variations and the storage time of the *E. coli*. The latter was confirmed in UV-Vis analysis experiments (see below) that even one day of cultivation duration had an impact on the outflow of NAD(P)^+^/NAD(P)H cofactor and proteins. Therefore, we compared the ratio of characteristic AC voltage, i.e. $AC \text {ratio}=\frac {U_{\mathrm {AC,inc}}}{U_{AC,0}}$ with *U*_AC,0_ AC voltage determined for the untreated cells and *U*_AC,inc_ AC voltage determined after incubation in isopropyl alcohol (Fig. 4) to study the impact of isopropyl alcohol on *E. coli*.

In Fig. 4, two different responses are visible. First, higher voltages were needed to trap 50% of the *E. coli*, i.e. *AC* ratio > 1, after incubation in 5% isopropyl alcohol even for incubation duration of 5 min. This indicates that the cells were less polarisable after incubation in isopropyl alcohol. This was further intensified for longer incubation durations. Second, the characteristic voltage decreased after incubation in 10% or 15% isopropyl alcohol, i.e. *AC* ratio < 1. Thus, the cells were better polarisable after incubation in higher concentrated isopropyl alcohol. These observations indicate that, at least, two different processes took part during incubation in isopropyl alcohol affecting the polarisability of *E. coli*.

We have to closer look to the physical mechanisms that have an effect on the dipole formation to understand the observed results of the DEP analysis. As described in the theory section, an electric dipole is induced in the *E. coli* by the electric field. The dipole moment is proportional to the polarisability of the object; i.e., the ability that its (free) charges can be spatially separated [36, 40]. Inter alia, this relies on the amount of (charged) molecules and proteins in cells and their close vicinity, e.g. the surrounding counter-ion cloud due to the negative surface charge [41]; Crowther et al. just recently demonstrated that small changes of the inner or outer membrane of *E. coli* affects the electrokinetic migration [42]. Additionally, the polarisability of cells depends on the charges on the cell surface and in the interior, as reported by Gyurova and Zhivkov, in the case of a Maxwell-Wagner polarisability [14]. Thus, we assume that if the cell membrane is permeabilised by incubation in isopropyl alcohol and charged molecules like small ions, cofactors, or proteins flow out of the cell, the amount of charges decreases and so does the polarisability. This could explain the behaviour observed for incubation in 5% isopropyl alcohol. Since leaking is diffusion-driven, it is a slow process that lasts over a longer time. Therefore, the increasing AC voltage for longer incubation durations is plausible. Gyurova and Zhivkov reported that the polarisability of *E. coli* decreased after incubation in ethanol as well, though, in contrast to our work, their measurement buffer contained ethanol [14]. Therefore, the dielectric properties of the surrounding medium were different for their control measurement and experiment. They explained the observed behaviour with the Maxwell-Wagner theory of polarisation, i.e. the polarisability of the *E. coli* is due to accumulation of ions in the cytoplasm in the vicinity of the cytoplasm membrane [14].

The effect that leads to the second behaviour, i.e. the increased polarisability, is assumed to be due to the impact of organic solvents on cell membrane fluidity. From literature, it is known that organic solvents integrate into the cell membrane [37], and thus lead to replacement of phospholipids [43] and a change of the membrane fluidity [37–39]. Therefore, the ability of membrane molecules to diffuse laterally in the membrane is increased. This could explain the higher polarisability of the cells incubated in 10% or 15% isopropyl alcohol. AFM (atomic force microscopy) images were taken from cells (before and after incubation in isopropyl alcohol (15% and 40%) for 30 min). No significant changes in the membrane morphology could be determined (data not shown).

To summarise the DEP analysis, we observed that already incubation in 5% isopropyl alcohol for 5 min leads to a detectable impact on the characteristic trapping voltage; a higher voltage was needed. Additionally, after incubation in isopropyl alcohol of higher concentrations, i.e. 10% and 15%, lower AC voltages were needed to trap the cells. Thus, the method provides insight into biophysical mechanisms, and thus better understanding of the processes taking part. For instance, distinguishing between slow, and thus long lasting processes as diffusion-driven release of small ions out of the cell, observed for 5% isopropyl alcohol, or fast processes, like incorporation of organic solvents into the membrane resulting in an increased fluidity, and thus higher polarisability.

### Absorbance analysis

In addition to the dielectrophoresis analysis of *E. coli* cells treated with isopropyl alcohol, we studied a second way to determine the influence of the organic solvent on the stability of the bacterial cells. In analogy to the dielectrophoresis experiments, we exposed the *E. coli* cells to different isopropyl alcohol concentrations (here 0–40%) for different times (here 5 min, 15 min, 30 min, 45 min). Afterwards, we separated the cells and determined the amount of NAD(P)^+^/NAD(P)H cofactor and protein released from the cells in the supernatant. The amount of protein was determined by standard Bradford protein determination assay [34] and the NAD(P)^+^/NAD(P)H cofactor concentration by a spectrophotometric assay determining the total NAD(P)^+^ and NAD(P)H concentration. Reaction conditions were chosen carefully to prevent damage of cells by other factors; e.g., the centrifugal speed was chosen low (5000 × *g*) and resuspension of cells was done by careful swirling or mild shaking.

**Fig. 5:**
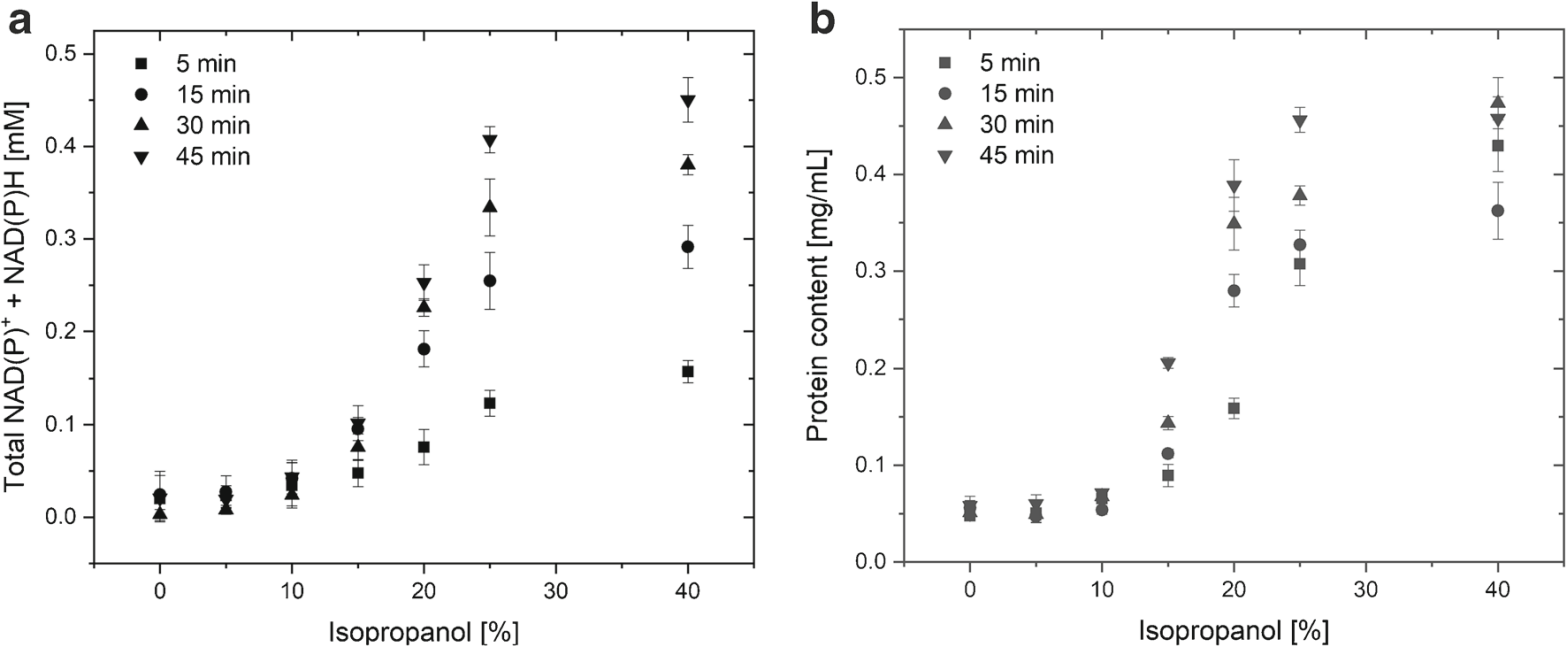
UV-Vis absorbance results. The cofactor (NAD(P)H) (**a**) and protein (**b**) content in the supernatant were measured after cell incubation in isopropyl alcohol of varying concentrations

As Fig. 5 shows, the release of NAD(P)^+^/NAD(P)H cofactor and protein from the cells can be monitored already at low isopropyl alcohol concentration around 10%. Starting from 15%, the release is well differentiable against the incubation without isopropyl alcohol, and increases further up to the treatment with 40% solvent. Longer incubation times increase the release of NAD(P)^+^/NAD(P)H cofactor and of protein, while the trend of release is the same for NAD(P)^+^/NAD(P)H cofactor and protein (Fig. 5a, b). During setup of the experimental procedure, we noticed that the quality of the cells has a high impact on the susceptibility of the cells, which is dependent on the storage time; already after 1 day in the fridge, the cells were more sensitive against the solvent; therefore, we performed the experiments with freshly prepared cells.

We determined experimentally the maximum content of NAD(P)^+^/NAD(P)H cofactor and protein by disruption of the same amount of cells (400 *μ* L of OD_600nm_= 20) by ultrasound sonication. This cell disruption method has the advantage that no additive like lysozyme is necessary so that volume and protein content are not manipulated. We observed a total NAD(P)^+^/NAD(P)H cofactor amount of 0.6 mM and a total protein amount of 1.3 mg/mL for this cell sample. This means that at 40% isopropyl alcohol, after 30-min incubation time, 63% (0.38 mM) of the NAD(P)^+^/NAD(P)H cofactor and 36% (0.47 mg/mL) of total protein are released. This indicates that the treatment of the cells with the isopropyl alcohol releases NAD(P)^+^/NAD(P)H cofactor in a higher amount than the protein which is expected as the cofactor is much smaller. Still, it should be considered that these numbers give a trend but should not be taken as absolute values as some protein might be bound to the membrane and thus could not be detected.

In contrast to the DEP method, this UV-Vis-based method detects the release of protein and cofactor from a bulk amount of cells and is thus more indirect. It is not possible to monitor individual cells as they cannot be prepared and trapped like in the DEP experiment, and the amount of released protein or cofactor would be too low to be determined. As the results show, the DEP method is much more sensitive in the low concentration range and can well differentiate between 5 and 10% isopropyl alcohol, where the absorbance analysis method reaches its limit. Small ions are not detected with that method but it is very likely that those are outflowing of the cell due to the isopropyl alcohol incubation and causing the change in the DEP trapping observed for the 5% isopropyl alcohol concentration.

## Conclusions

We have demonstrated a fast analysis method to study the impact of isopropyl alcohol on *E. coli* by means of dielectrophoresis. The analysis revealed that already incubation in 5% isopropyl alcohol for 5 min leads to a significant change of the DEP migration behaviour; the polarisability decreased compared to the reference cells. Additionally, we could demonstrate that incubation in isopropyl alcohol of higher concentration, i.e. 10% and 15%, led to increase of the polarisability. Therefore, we conclude that (at least) two different processes took part during the incubation process that led to these two different responses for the different isopropyl alcohol concentrations.

Besides the new DEP analysis, we also conducted established UV-Vis absorbance measurements to determine the amount of proteins and NAD(P)^+^/NAD(P)H cofactors that were released into the surrounding medium as complementary experiments. The method can differentiate between the released NAD(P)H and protein at different isopropyl alcohol concentrations, especially above 15% isopropyl alcohol. The UV-Vis experiments confirmed that the solvent treatment does lead to a release of inner cell components, but with the current method it will hardly be possible to study the molecular reasons and to differentiate changes in cell response at low isopropyl alcohol concentration around 5% or 10%.

The results of both measurement approaches were in good agreement, though the novel method by dielectrophoresis analysis provided a more sensitive approach with insight into the processes that take place during incubation with isopropyl alcohol. For example, the DEP analysis offers access to distinguish between fast processes, like the incorporation of organic solvents into the membrane and slow processes as the diffusion-driven release of cell compounds, e.g. cofactors, out of the cell. Hence the presented DEP analysis method is a versatile tool for further studies of the impact of organic solvents on whole-cell biocatalysts.

## Electronic supplementary material

Below is the link to the electronic supplementary material.

(PDF 608 KB)
