# Supplementary material for: Dielectrophoretic analysis of the impact of isopropyl alcohol on the electric polarisability of Escherichia coli whole-cells
Source: Anal Bioanal Chem. 2020 Mar 11;412(16):3925–33. doi: 10.1007/s00216-020-02451-9 (PMC7235074; doi:10.1007/s00216-020-02451-9)
Supplement: Supplementary file 1 — (PDF 608 KB) [file 216_2020_2451_MOESM1_ESM.pdf]

Analytical and Bioanalytical Chemistry

Electronic Supplementary Material

Dielectrophoretic analysis of the impact of isopropyl alcohol on the electric polarisability of *Escherichia coli* whole cells

Miriam S. Epping, Severin Wedde, Armin Grundmann, Marco Radukic, Harald Gröger, Anke Hummel, Martina Viefhues

All chemicals were obtained from Carl Roth or VWR, all molecular biological tools from Thermo Fisher Scientific, unless otherwise stated. The gene of the fluorescence protein SuperfolderGFP was codon-optimized with the GeneOptimizer^{TM} algorithm [1] for expression in *Escherichia coli*. For periplasmic expression the ompA signal sequence [2] from *E. coli* in its native codon-usage was N-terminally fused to the SuperfolderGFP-gene. For regulation of transcription the strong T5-promoter [3] - recognized by the *E. coli* RNA polymerase [4] and thus applicable independent of the used *E. coli* strain - with lac-operator and lambda T0 transcriptional termination region was chosen. Translational strength was modulated by the strong ribosome binding site (RBS) AGGAGA and an optimal distance between RBS and start codon of 8 nucleotides [5] The complete expression cassette was obtained as synthetic DNA from Thermo Fisher Scientific GmbH with SgRAI restriction site at the 5'-end and BlpI restriction site at the 3'end for sticky end insertion in pET-21a(+) (Novagen/Merck KGaA). The obtained plasmid was named pET21T5 ompA-co-SuperfolderGFP(-).

For cytoplasmic expression of the SuperfolderGFP, the sequence of the N-terminal ompA signal peptide had to be removed from pET21T5 ompA-co-SuperfolderGFP(-). Therefore, the complete plasmid was amplified by gradient PCR while leaving out the ompA signal sequence using forward primer 5'-CATAGTTAATTTCTCCTCTTTAATGAATTC-3' and reverse primer:

5'-AGCAAAGGCGAAGAACTG-3' (Eurofins Genomics Deutschland GmbH). The master mix consisted of 120.6 µL water, 32 µL 5x Phusion HF-buffer, 3.2 µL 10 mM dNTPs, 0.8 µL forward primer (10 µM), 0.8 µL reverse primer (10 µM), 1 µL of 1 ng/µL pET21T5 ompA-co-SuperfolderGFP(-) as template and 1.6 µL Phusion H.-F. DNA-polymerase. Aliqots of 20 µL were placed in a row of the PCR cycler (SensoQuest GmbH) applying the following temperature profile for DNA-amplification: initial denaturation for 3 min at 98 °C, 35 repetitive cycles consisting of a denaturation step at 98 °C for 30 s, an annealing step for 30 s with a temperature gradient of 56-70 °C and an elongation step at 72 °C for 3 min and 6 s, followed by a final elongation step at 72 °C for 4 min.

Amplification and specificity of PCR was verified on a 1 % agarose gel, prepared from 300 mg agarose NEEO ultra-quality dissolved and heated up in 30 mL 0.5x TAE (2.4 g/L Tris, 0.5 mL/L acetic acid (VWR International GmbH), 185 mg/L Na2EDTA, pH 8.0). After cooling to 60 °C 5 µL Roti-Safe were added for visualization of DNA under UV-light. 10 µL of each reaction mixture was mixed with 2 µL of 6x loading dye (10 mM Tris-HCl, 0.03 % bromophenol blue, 0.03 % xylene cyanol FF (Sigma Aldrich), 60 % glycerol, 60 mM EDTA, pH 7.6) alongside 5 µL GeneRuler 1 kb DNA Ladder as size marker were

loaded on the gel and separated for 60 min at 100 V. Bands corresponding to the correct fragment having a size of 6,163 bps were cut with a scalpel (B. Braun Melsungen AG) and transferred to pre-weighted 2 mL reaction tube (Eppendorf AG). DNA was isolated with Wizard SV Gel and PCR Clean-Up System (Promega GmbH) according to the manufacturer's protocol.

Subsequent 100 ng of purified PCR product were phosphorylated by adding 2 µL 10x FastDigest ^{R} buffer with 10 mM ATP, 1 µL T4-polynucleotide kinase in a volume of 20 µL (filled up with water) at 37 °C for 20 min. PNK was heat inactivated for 5 min at 70 °C. For ligation of the ends to a circular plasmid 17 µL water, 3 µL 10x FastDigest ^{R} buffer with 10 mM ATP, 5 µL PEG 4000 and 5µL T4-ligase were added. Reaction mixture was incubated for 16 h at room temperature. Ligase was inactivated for 5 min at 70 °C. Template DNA was removed by addition of 0.5 µL DpnI and incubation for 2 h at 37 °C. DpnI was inactivated by incubating for 20 min at 80 °C. 5 µL reaction mixture were transformed in chemical competent cells according to the heat shock method of Cohen et al. [6] and plated on LB agar plates (10 g/L NaCl, 10 g/L peptone, 5 g/L yeast extract, 15 g/L agar-agar, pH 7.4) containing 100 mg/L carbenicillin. A few clones were grown in 5 mL LB medium [7] supplemented with 100 mg/L carbenicillin in a culture tube over night at 37 °C at 180 rpm. Plasmids were isolated with Wizard Plus SV Minipreps DNA Purification System (Promega GmbH) according to the manufacturer's protocol. 15 µL plasmid DNA (50-100 ng/µL) and 2 µL either sequencing primer sequencing primer forward: 5'-GTGATGTCGGCGATATAG-3' (10 µM) or sequencing primer reverse: 5'-TCAGCCTAGCTTGGATTC-3' (10 µM) (Eurofins Genomics Deutschland GmbH) were mixed and were send to Eurofins Genomics Deutschland GmbH for sequencing, which confirmed the correct cloning of pET21T5 co-SuperfolderfGFP(-).

Coding sequence of SuperfolderGFP with optimized codon usage for expression in *E. coli* is as follows:

ATGAGCAAAGGCGAAGAACTGTTTACCGGTGTTGTTCCGATTCTGGTTGAACTGGATGGTGATGTTAATGGCACAAATTTT
CAGTTCGTGGTGAAGGCGAAGGTGATGCAACCAATGGTAAACTGACCCTGAAATTTATCTGTACCACCGGCAAACTGCCG
GTTCCGTGGCCGACCCTGGTTACCACCCTGACCTATGGTGTTCAGTGTTTTAGCCGTTATCCGGATCATATGAAACAGCACG
ATTTTTTCAAAAGCGCAATGCCGGAAGGTTATGTTCAAGAACGTACCATCTCCTTTAAAGATGATGGCACCTATAAAACCC
GTGCCGAAGTTAAATTTGAAGGTGATACCCTGGTGAATCGCATTGAACTGAAAGGCATCGATTTCAAAGAAGATGGTAAT
ATCCTGGGCCACAAACTGGAATATAATTTCAATAGCCACAACGTGTATATCACCGCAGACAAACAGAAAAATGGCATCAAA
GCCAACTTTAAAATCCGGCATAATGTTGAAGATGGCAGCGTTCAGCTGGCAGATCATTATCAGCAGAATACCCCGATTGGT
GATGGTCCGGTTCTGCTGCCGGATAATCATTATCTGAGCACCCAGAGCGTTCTGAGCAAAGATCCGAATGAAAAACGTGA
TCACATGGTGCTGCTGGAATTTGTTACCGCAGCAGGTATTACCCATGGTATGGATGAACTGTACAAATAA

Amino acid sequence of SuperfolderGFP is as follows:

MSKGEELFTGVVPILVELDGDVNGHKFSVRGEGEGDATNGKLTLKFICTTGKLPVPWPTLVTTLTYGVQCFSRYPDHMKQHDFF
KSAMPEGYVQERTISFKDDGTYKTRAEVKFEGDTLVNRIELKGIDFKEDGNILGHKLEYNFNSHNVYITADKQKNGIKANFKIRHN
VEDGSVQLADHYQQNTPIGDGPVLLPDNHYLSTQSVLSKDPNEKRDHMVLLEFVTAAGITHGMDELYK

Vector map and complete sequence of the expression plasmid pET21T5_co-SuperfolderfGFP(-) are shown in the following:

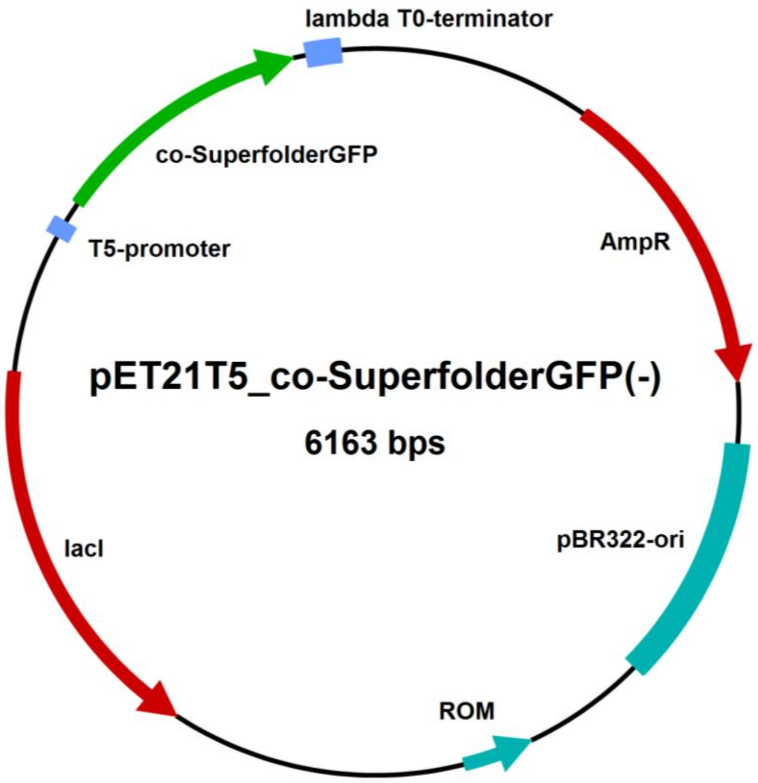

ATCCGGATATAGTTCCTCCTTTCAGCAAAAAACCCCTCAAGACCCGTTTAGAGGCCCCAAGGGGTTATGCTAGTTATTGCTC
AGCCTAGCTTGGATTCTCACCAATAAAAAACGCCCGGCGGCAACCGAGCGTTCTGAACAAATCCAGATGGAGTTCTGAGG
TCATTACTGGATCTATCAACAGGAGTCCAAGCTCAGTCATTAGTGATGGTGATGGTGATTCATTATTTGTACAGTTCATCCA
TACCATGGGTAATACCTGCTGCGGTAACAAATTCCAGCAGCACCATGTGATCACGTTTTTCATTCGGATCTTTGCTCAGAAC
GCTCTGGGTGCTCAGATAATGATTATCCGGCAGCAGAACCGGACCATCACCAATCGGGGTATTCTGCTGATAATGATCTGC
CAGCTGAACGCTGCCATCTTCAACATTATGCCGGATTTTAAAGTTGGCTTTGATGCCATTTTTCTGTTTGTCTGCGGTGATAT
ACACGTTGTGGCTATTGAAATTATATTCCAGTTTGTGGCCCAGGATATTACCATCTTCTTTGAAATCGATGCCTTTCAGTTCA
ATGCGATTCACCAGGGTATCACCTTCAAATTTAACTTCGGCACGGGTTTTATAGGTGCCATCATCTTTAAAGGAGATGGTAC
GTTCTTGAACATAACCTTCCGGCATTGCGCTTTTGAAAAAATCGTGCTGTTTCATATGATCCGGATAACGGCTAAAACACTG
AACACCATAGGTCAGGGTGGTAACCAGGGTCGGCCACGGAACCGGCAGTTTGCCGGTGGTACAGATAAATTTCAGGGTC
AGTTTACCATTGGTTGCATCACCTTCGCCTTCACCACGAACTGAAAATTTGTGGCCATTAACATCACCATCCAGTTCAACCA
GAATCGGAACAACACCGGTAAACAGTTCTTCGCCTTTGCTCATAGTTAATTTCTCCTCTTTAATGAATTCTGTGTGAAATTGT
TATCCGCTCACAATTGAATCTATTATAATTGTTATCCGCTCACAAAGCAAATAAATTTTTTATGATTTCTCGAGGTGAAGACG
AAAGGGCCTCGTGATACGCCTATTTTTATAGGTTAACACCGGCGCCACAGGTGCGGTTGCTGGCGCCTATATCGCCGACAT
CACCGATGGGGAAGATCGGGCTCGCCACTTCGGGCTCATGAGCGCTTGTTTCGGCGTGGGTATGGTGGCAGGCCCCGTG
GCCGGGGGACTGTTGGGCGCCATCTCCTTGCATGCACCATTCCTTGCGGCGGCGGTGCTCAACGGCCTCAACCTACTACTG
GGCTGCTTCCTAATGCAGGAGTCGCATAAGGGAGAGCGTCGAGATCCCGGACACCATCGAATGGCGCAAAACCTTTCGCG
GTATGGCATGATAGCGCCCGGAAGAGAGTCAATTCAGGGTGGTGAATGTGAAACCAGTAACGTTATACGATGTCGCAGA
GTATGCCGGTGTCTCTTATCAGACCGTTTCCCGCGTGGTGAACCAGGCCAGCCACGTTTCTGCGAAAACGCGGGAAAAAG
TGGAAGCGGCGATGGCGGAGCTGAATTACATTCCCAACCGCGTGGCACAACAACTGGCGGGCAAACAGTCGTTGCTGATT
GGCGTTGCCACCTCCAGTCTGGCCCTGCACGCGCCGTCGCAAATTGTCGCGGCGATTAAATCTCGCGCCGATCAACTGGGT
GCCAGCGTGGTGGTGTCGATGGTAGAACGAAGCGGCGTCGAAGCCTGTAAAGCGGCGGTGCACAATCTTCTCGCGCAAC
GCGTCAGTGGGCTGATCATTAACTATCCGCTGGATGACCAGGATGCCATTGCTGTGGAAGCTGCCTGCACTAATGTTCCGG
CGTTATTTCTTGATGTCTCTGACCAGACACCCATCAACAGTATTATTTTCTCCCATGAAGACGGTACGCGACTGGGCGTGGA
GCATCTGGTCGCATTGGGTCACCAGCAAATCGCGCTGTTAGCGGGCCCATTAAGTTCTGTCTCGGCGCGTCTGCGTCTGGC
TGGCTGGCATAAATATCTCACTCGCAATCAAATTCAGCCGATAGCGGAACGGGAAGGCGACTGGAGTGCCATGTCCGGTT
TTCAACAAACCATGCAAATGCTGAATGAGGGCATCGTTCCCACTGCGATGCTGGTTGCCAACGATCAGATGGCGCTGGGC
GCAATGCGCGCCATTACCGAGTCCGGGCTGCGCGTTGGTGCGGATATCTCGGTAGTGGGATACGACGATACCGAAGACA
GCTCATGTTATATCCCGCCGTTAACCACCATCAAACAGGATTTTCGCCTGCTGGGGCAAACCAGCGTGGACCGCTTGCTGC

AACTCTCTCAGGGCCAGGCGGTGAAGGGCAATCAGCTGTTGCCCGTCTCACTGGTGAAAAGAAAAACCACCCTGGCGCCC
AATACGCAAACCGCCTCTCCCCGCGCGTTGGCCGATTCATTAATGCAGCTGGCACGACAGGTTTCCCGACTGGAAAGCGG
GCAGTGAGCGCAACGCAATTAATGTAAGTTAGCTCACTCATTAGGCACCGGGATCTCGACCGATGCCCTTGAGAGCCTTCA
ACCCAGTCAGCTCCTTCCGGTGGGCGCGGGGCATGACTATCGTCGCCGCACTTATGACTGTCTTCTTTATCATGCAACTCGT
AGGACAGGTGCCGGCAGCGCTCTGGGTCATTTTCGGCGAGGACCGCTTTCGCTGGAGCGCGACGATGATCGGCCTGTCGC
TTGCGGTATTCGGAATCTTGCACGCCCTCGCTCAAGCCTTCGTCACTGGTCCCGCCACCAAACGTTTCGGCGAGAAGCAGG
CCATTATCGCCGGCATGGCGGCCCCACGGGTGCGCATGATCGTGCTCCTGTCGTTGAGGACCCGGCTAGGCTGGCGGGGT
TGCCTTACTGGTTAGCAGAATGAATCACCGATACGCGAGCGAACGTGAAGCGACTGCTGCTGCAAAACGTCTGCGACCTG
AGCAACAACATGAATGGTCTTCGGTTTCCGTGTTTCGTAAAGTCTGGAAACGCGGAAGTCAGCGCCCTGCACCATTATGTT
CCGGATCTGCATCGCAGGATGCTGCTGGCTACCCTGTGGAACACCTACATCTGTATTAACGAAGCGCTGGCATTGACCCTG
AGTGATTTTTCTCTGGTCCCGCCGCATCCATACCGCCAGTTGTTTACCCTCACAACGTTCCAGTAACCGGGCATGTTCATCAT
CAGTAACCCGTATCGTGAGCATCCTCTCTCGTTTCATCGGTATCATTACCCCCATGAACAGAAATCCCCCTTACACGGAGGC
ATCAGTGACCAAACAGGAAAAAACCGCCCTTAACATGGCCCGCTTTATCAGAAGCCAGACATTAACGCTTCTGGAGAAACT
CAACGAGCTGGACGCGGATGAACAGGCAGACATCTGTGAATCGCTTCACGACCACGCTGATGAGCTTTACCGCAGCTGCC
TCGCGCGTTTCGGTGATGACGGTGAAAACCTCTGACACATGCAGCTCCCGGAGACGGTCACAGCTTGTCTGTAAGCGGAT
GCCGGGAGCAGACAAGCCCGTCAGGGCGCGTCAGCGGGTGTTGGCGGGTGTCGGGGCGCAGCCATGACCCAGTCACGT
AGCGATAGCGGAGTGTATACTGGCTTAACTATGCGGCATCAGAGCAGATTGTACTGAGAGTGCACCATATATGCGGTGTG
AAATACCGCACAGATGCGTAAGGAGAAAATACCGCATCAGGCGCTCTTCCGCTTCCTCGCTCACTGACTCGCTGCGCTCGG
TCGTTCGGCTGCGGCGAGCGGTATCAGCTCACTCAAAGGCGGTAATACGGTTATCCACAGAATCAGGGGATAACGCAGG
AAAGAACATGTGAGCAAAAGGCCAGCAAAAGGCCAGGAACCGTAAAAAGGCCGCGTTGCTGGCGTTTTTCCATAGGCTC
CGCCCCCCTGACGAGCATCACAAAAATCGACGCTCAAGTCAGAGGTGGCGAAACCCGACAGGACTATAAAGATACCAGGC
GTTTCCCCCTGGAAGCTCCCTCGTGCGCTCTCCTGTTCCGACCCTGCCGCTTACCGGATACCTGTCCGCCTTTCTCCCTTCGG
GAAGCGTGGCGCTTTCTCATAGCTCACGCTGTAGGTATCTCAGTTCGGTGTAGGTCGTTCGCTCCAAGCTGGGCTGTGTGC
ACGAACCCCCCGTTCAGCCCGACCGCTGCGCCTTATCCGGTAACTATCGTCTTGAGTCCAACCCGGTAAGACACGACTTATC
GCCACTGGCAGCAGCCACTGGTAACAGGATTAGCAGAGCGAGGTATGTAGGCGGTGCTACAGAGTTCTTGAAGTGGTGG
CCTAACTACGGCTACACTAGAAGGACAGTATTTGGTATCTGCGCTCTGCTGAAGCCAGTTACCTTCGGAAAAAGAGTTGGT
AGCTCTTGATCCGGCAAACAAACCACCGCTGGTAGCGGTGGTTTTTTTGTTTGCAAGCAGCAGATTACGCGCAGAAAAAA
AGGATCTCAAGAAGATCCTTTGATCTTTTCTACGGGGTCTGACGCTCAGTGGAACGAAAACTCACGTTAAGGGATTTTGGT
CATGAGATTATCAAAAAGGATCTTCACCTAGATCCTTTTAAATTAAAAATGAAGTTTTAAATCAATCTAAAGTATATATGAG
TAAACTTGGTCTGACAGTTACCAATGCTTAATCAGTGAGGCACCTATCTCAGCGATCTGTCTATTTCGTTCATCCATAGTTGC
CTGACTCCCCGTCGTGTAGATAACTACGATACGGGAGGGCTTACCATCTGGCCCCAGTGCTGCAATGATACCGCGAGACCC
ACGCTCACCGGCTCCAGATTTATCAGCAATAAACCAGCCAGCCGGAAGGGCCGAGCGCAGAAGTGGTCCTGCAACTTTAT
CCGCCTCCATCCAGTCTATTAATTGTTGCCGGGAAGCTAGAGTAAGTAGTTCGCCAGTTAATAGTTTGCGCAACGTTGTTGC
CATTGCTGCAGGCATCGTGGTGTCACGCTCGTCGTTTGGTATGGCTTCATTCAGCTCCGGTTCCCAACGATCAAGGCGAGT
TACATGATCCCCCATGTTGTGCAAAAAAGCGGTTAGCTCCTTCGGTCCTCCGATCGTTGTCAGAAGTAAGTTGGCCGCAGT
GTTATCACTCATGGTTATGGCAGCACTGCATAATTCTCTTACTGTCATGCCATCCGTAAGATGCTTTTCTGTGACTGGTGAG
TACTCAACCAAGTCATTCTGAGAATAGTGTATGCGGCGACCGAGTTGCTCTTGCCCGGCGTCAATACGGGATAATACCGCG
CCACATAGCAGAACTTTAAAAGTGCTCATCATTGGAAAACGTTCTTCGGGGCGAAAACTCTCAAGGATCTTACCGCTGTTG
AGATCCAGTTCGATGTAACCCACTCGTGCACCCAACTGATCTTCAGCATCTTTTACTTTCACCAGCGTTTCTGGGTGAGCAA
AAACAGGAAGGCAAAATGCCGCAAAAAAGGGAATAAGGGCGACACGGAAATGTTGAATACTCATACTCTTCCTTTTTCAA
TATTATTGAAGCATTTATCAGGGTTATTGTCTCATGAGCGGATACATATTTGAATGTATTTAGAAAAATAAACAAATAGGG
GTTCCGCGCACATTTCCCCGAAAAGTGCCACCTGAAATTGTAAACGTTAATATTTTGTTAAAATTCGCGTTAAATTTTTGTTA
AATCAGCTCATTTTTTAACCAATAGGCCGAAATCGGCAAAATCCCTTATAAATCAAAAGAATAGACCGAGATAGGGTTGAG
TGTTGTTCCAGTTTGGAACAAGAGTCCACTATTAAAGAACGTGGACTCCAACGTCAAAGGGCGAAAAACCGTCTATCAGG
GCGATGGCCCACTACGTGAACCATCACCCTAATCAAGTTTTTTGGGGTCGAGGTGCCGTAAAGCACTAAATCGGAACCCTA
AAGGGAGCCCCCGATTTAGAGCTTGACGGGGAAAGCCGGCGAACGTGGCGAGAAAGGAAGGGAAGAAAGCGAAAGGA
GCGGGCGCTAGGGCGCTGGCAAGTGTAGCGGTCACGCTGCGCGTAACCACCACACCCGCCGCGCTTAATGCGCCGCTACA
GGGCGCGTCCCATTCGCCA

References

[1] D. Raab, M. Graf, F. Notka, T. Schödl, R. Wagner, Systems and synthetic biology 4(3), 215 (2010)

[2] N.R. Movva, K. Nakamura, M. Inouye, Journal of Biological Chemistry 255(1), 27 (1980)

[3] L. Vidal, P. Ferrer, G. Alvaro, M.D. Benaiges, G. Caminal, Journal of Biotechnology 118(1), 75 (2005)

[4] R. Gentz, H. Bujard, Journal of bacteriology 164(1), 70 (1985)

[5] S. Ringquist, S. Shinedling, D. Barrick, L. Green, J. Binkley, G.D. Stormo, L. Gold, Molecular microbiology 6(9), 1219 (1992)

[6] S.N. Cohen, A.C. Chang, L. Hsu, Proceedings of the National Academy of Sciences 69(8), 2110 (1972)

[7] G. Bertani, Journal of bacteriology 62(3), 293 (1951)
